# Empirical comparison of time series models and tensor product penalised splines for modelling spatial dependence in plant breeding field trials

**DOI:** 10.3389/fpls.2022.1021143

**Published:** 2023-01-18

**Authors:** Beverley Gogel, Sue Welham, Brian Cullis

**Affiliations:** ^1^ Centre for Biometrics and Data Science for Sustainable Primary Industries, National Institute for Applied Statistics Research Australia, School of Mathematics and Applied Statistics, University of Wollongong, Wollongong, NSW, Australia; ^2^ Stats4Biol Consultancy Limited, Welwyn Garden City, United Kingdom

**Keywords:** ARIMA time series models, tensor product penalised spline, separable lattice process, spatial dependence, linear mixed models, genetic relatedness, Akaike information criteria

## Abstract

Plant breeding field trials are typically arranged as a row by column rectangular lattice. They have been widely analysed using linear mixed models in which low order autoregressive integrated moving average (ARIMA) time series models, and the subclass of separable lattice processes, are used to account for two-dimensional spatial dependence between the plot errors. A separable first order autoregressive model has been shown to be particularly useful in the analysis of plant breeding trials. Recently, tensor product penalised splines (TPS) have been proposed to model two-dimensional smooth variation in field trial data. This represents a non-stochastic smoothing approach which is in contrast to the autoregressive (AR) approach which models a stochastic covariance structure between the lattice of errors. This paper compares the AR and TPS methods empirically for a large set of early generation plant breeding trials. Here, the fitted models include information on genetic relatedness among the entries being evaluated. This provides a more relevant framework for comparison than the assumption of independent genetic effects. Judged by Akaike Information Criteria (AIC), the AR models were a better fit than the TPS model for more than 80% of trials. In the cases where the TPS model provided a better fit it did so by only a small amount whereas the AR models made a substantial improvement across a range of trials. When the AR and TPS models differ, there can be marked differences in the ranking of genotypes between the two methods of analysis based on their predicted genetic effects. Using the best fitting model for a trial as the benchmark, the rate of mis-classification of entries for selection was greater for the TPS model than the AR models. This has important practical implications for breeder selection decisions.

## 1 Introduction

Spatial dependence between neighbouring plots occurs naturally in plant breeding and other agricultural field trials laid out as a row by column rectangular lattice. This is due to heterogeneity across the trial area, mostly due to local variation in soil conditions, for example, changes in soil fertility and moisture content. As early as the 1920’s, Sir R. A. Fisher was concerned that yield observations on adjacent plots in field trials were highly correlated relative to observations that were further apart (Box, 1978). Since then there is a rich literature on methods to account for this dependence in field trial analysis, where a main aim is to produce accurate and reliable estimates of the treatments under evaluation (or treatment contrasts) and their standard errors. Early methods involved adjusting plot yields by the value on neighbouring plots in a covariance analysis (Papadakis, 1937; Bartlett, 1978). Differencing techniques were introduced to remove non-stationary spatial association between adjacent plots in a single dimension (Wilkinson et al. (1983); Green et al. (1985); Green (1985); Besag and Kempton (1986)), and Gleeson and Cullis (1987) modelled spatial correlation directly using a low order autoregressive integrated moving average (ARIMA) process, also in one dimension. Their framework was a linear mixed model (LMM) for first- or second-differenced data and they used the residual maximum likelihood (REML) method (Patterson and Thompson, 1971) for variance parameter estimation. Martin (1990) encouraged the use of time series models for field trial data and suggested that the subclass of separable lattice processes could overcome many of the problems associated with modelling covariance in two dimensions. Motivated by Martin (1990); Cullis and Gleeson (1991) extended their one-dimensional spatial analysis to a two dimensional analysis assuming separability. Martin (1990) recognised that increased correlation along rows and columns due to trial management practices was a common cause of non-stationarity in field trial data, and Cullis and Gleeson (1991) recommended differencing the data to ensure that the spatial process is stationary. However, empirical evidence over many years suggests that a separable first order autoregressive process (A×A), together with random row and column terms, generally provides an adequate fit for (un-differenced) field trial data measured on a rectangular lattice. Where the A×A model does not provide an adequate representation of the spatial dependence in the data, a wide range of alternative models are available through the ARIMA class. This provides a rich and very flexible framework for modelling two-dimensional spatial dependence.

A simple extension of the A×A model is the errors-in-variables model of Besag (1977) which adds an independent error or uncorrelated noise component to the spatial process. He explained that this formulation might be appropriate for agricultural field trial data where “*plant yields are likely to reflect local variations in soil fertility as well as intrinsic variability in the plants themselves”*. We label this the A×Ae model.

The spatial methods of Cullis and Gleeson (1991) have been in widespread use since they were first introduced in the 1990’s (see for example, Smith et al. (2001); Ganesalingam et al. (2012); Oakey et al. (2016); De Faveri et al. (2017); Norman et al. (2018); Hunt et al. (2020). Gilmour et al. (1997) popularised the method and suggested the use of residual diagnostics to identify non-stationary effects across the trial area and to assist in identifying an appropriate spatial model for the plot errors. Recently, tensor product penalised splines (TPS) have been implemented to explicitly model smooth variation in field trial data (Rodríguez-Álvarez et al., 2017) They are fitted as a two-dimensional smooth surface within an LMM in which the terms that form the smooth surface account for both small- and large-scale continuous trend effects across the trial region. This is a non-stochastic method (no assumed dependence structure) and is in contrast to the ARIMA method which models a separable and spatially correlated stochastic error process for the lattice or residuals, with additional terms to ensure that the assumption of stationarity is met.

The TPS model can be accessed through the SpATS package (Rodríguez-Álvarez et al., 2017) in R (R Development Core Team, 2020). Velazco et al. (2017) compare the A×Ae model to the TPS model for a set of Australian sorghum breeding trials and Rodríguez-Álvarez et al. (2017) provide a comparison under simulation. They used the SpATS package for the TPS model and the ASReml-R software (Butler et al., 2018) for the A×Ae model. Currently the SpATS package is limited to the assumption of independent and identically distributed (IID) random genetic effects. The TPSbits package (Welham, 2020) has recently been developed to allow us to fit the TPS model using ASReml-R and in LMM that incorporate genetic-relatedness through either ancestral (pedigree) information or genomic (molecular marker) data. This has allowed us to compare the A×A, A×Ae (collectively AR) and TPS models, as well as a baseline model which represents no modelling of spatial dependence, for LMM that include more plausible models for the genetic effects.

We have compared the AR, TPS and baseline models for a set of 110 pulse breeding trials. To a degree this work has been motivated by Laslett (1994) who examined the difference between the kriging method (stochastic) and the spline method (non-stochastic) for spatial prediction from both grid data and sparse samples in a geostatistical context. He showed that “*for data sets that, from various diagnostics, appear to be a realization of a stationary stochastic process with autocovariance monotonically decreasing to 0 with increasing lag, kriging may outperform splines as a predictor by a factor of two or more in mean squared error of prediction…”*. This research is to investigate if similar results hold between stochastic and non-stochastic methods in this alternate setting.

The paper is arranged as follows. We first introduce the empirical data set, Section 2. The AR, TPS and baseline LMM are special cases of a general LMM which we present in Section 3. Technical details concerning the fitted LMM and comparisons between them are given in Section 4 and the results of the empirical study are presented in Section 5. A discussion is presented in Section 6.

## 2 Empirical data set

We considered a set of early generation lentil and field pea trials conducted by Agriculture Victoria between 2016 and 2020. The lentil data comprised 48 stage 0 (labelled S0), 1 (S1) and 2 (S2) trials conducted across 28 environments in South Australia and Victoria. The field pea data comprised 62 stage 0 (labelled P0), 1 (P1) and 2 (P2) trials conducted across 45 environments in Victoria, New South Wales, South Australia and Western Australia. Collectively, the lentil and field pea data are considered to be representative of early stage plant breeding field trial data in Australia. **Supplementary Table 1** gives a summary of the full set of 110 trials, each of which was laid out as a rectangular lattice with 12 columns and between 6 and 88 rows. With the exception of one trial (LGS0HO19), the lentil S0 and S1 trials were designed as p-rep trials (Cullis et al., 2006) with two replicate blocks in either one or both directions and a single occurrence of each replicated test entry in each replicate. The vast majority of test entries in these trials were unreplicated. LGS0HO19 was designed as a randomised complete block (RCB) trial with three blocks. The lentil S2 trials were designed as RCB trials with two replicates and blocking in a single direction for all but L2RHO18 which was blocked in both directions. For field peas, the P0 trials were designed as p-rep trials with blocking in a single direction and most test entries unreplicated. The P1 and P2 trials were designed as RCB trials with two replicates. We note that a small subset of test entries had just one replicate in a number of the S2, P1 and P2 trials, indicated in the single replicate (1r) column in the summary table. Prior to 2019, the lentil and field pea trials were designed by the breeding program in-house. From 2019, model based designs have been generated using the od software (Butler, 2013) available in R (R Development Core Team, 2020). Furthermore, the stage 0 and stage 1 trials for both crops have been designed using pedigree information (Cullis et al., 2020).

For the lentil data, pedigree information was available for 5771 individuals comprising the 5049 individuals in the full set of trials conducted between 2016 and 2020 (including both early and late stage trials) and 722 additional individuals (ancestors). For field peas, pedigree information was available for 11482 individuals comprising the 3511 individuals in the full set of trials and 7971 additional individuals. In the empirical study we utilized the full pedigree in the analysis of each trial within each crop.

## 3 Statistical methods

We have compared the performance of a baseline model that ignores spatial dependence with the A×A, A×Ae and TPS models. The baseline, A×A and A×Ae models fall within the ARIMA class of models. The TPS model is from a separate, non-stochastic class but can be considered within the same statistical framework.

### 3.1 General form of the LMM for field trial analysis

Let y be an *n*×1 vector of phenotypic data from an individual field trial ordered as rows within columns. An LMM for field trial analysis that accommodates the baseline, AR and TPS models has the following general form:


(1) $$y=X_{p}\tau_{p}+Z_{g}u_{g}+Z_{p}u_{p}+e$$

where $\tau_{p}$ is a *t*×1 vector of fixed effects with design matrix $X_{p}$ , $u_{g}$ is an *m*×1 vector of genetic effects with design matrix $Z_{g}$ , $u_{p}$ is a *q*×1 vector of non-genetic (or peripheral) random effects with design matrix $Z_{p}$ , and e is the *n*×1 vector of plot errors. We note that $\tau_{p}$ includes the overall mean and may include other fixed effects. We assume that $u_{g}$ , $u_{p}$ and e are mutually independent and have a multivariate normal distribution with zero means and covariance matrices $\text{var}(u_{g})=G_{g}(\sigma_{g})$ , $\text{var}(u_{p})=G_{p}(\sigma_{p})$ and $\text{var}(e)=R(\sigma_{r})$ , where $\sigma_{g}$ , $\sigma_{p}$ and $\sigma_{r}$ are sets of variance parameters for the genetic and peripheral effects and the plot errors, respectively.

#### 3.1.1 The genetic effects

In the case where information on genetic relatedness is included in the analysis, the effects in $u_{g}$ are written as the sum of a set of additive genetic effects ( $u_{a}$ ) and a set of non-additive (or residual) genetic effects ( $u_{e}$ ), that is,


$$u_{g}=u_{a}+u_{e}$$

The additive effects can be modelled using either pedigree information or marker data, see Tolhurst et al. (2019). Here we use pedigree information but the marker model takes a similar form. Let A be the *m*×*m* numerator relationship matrix (see Oakey et al. (2006) and Beeck et al. (2010), for example). In this case the variance matrix of the additive genetic effects is given by $\text{var}(u_{a})=\sigma_{a}^{2}A$ . The non-additive effects represent the specific performance of the individuals in the data that cannot be accounted for by their ancestry (Oakey et al., 2006). We assume that they are a set of IID effects, that is, $\text{var(}u_{e})=\sigma_{e}^{2}I_{m},$ and that $u_{a}$ and $u_{e}$ are mutually independent sets of effects. The covariance matrix for the total genetic effects is then


(2) $$G_{g}=\sigma_{a}^{2}A+\sigma_{e}^{2}I_{m}$$

#### 3.1.2 Non-genetic fixed and random effects and the errors

**Table 1** is a summary of the fixed and non-genetic random terms and the error variance structure for the four models (baseline, A×A, A×Ae, TPS) that were fitted to the yield data for each trial.

**Table 1:** Summary of the fixed and non-genetic random terms, and the error variance structure in the A×A, A×Ae, TPS and baseline LMM.

Model	Fixed (*X_p_*)	Non-genetic random (*Z_p_*)	*R*(*σ_r_*)	Total variance parameters
baseline	$1_{n}$	[ $Z_{b}\;Z_{\mathrm{row}}\;Z_{\mathrm{col}}$ ]	$\sigma^{2}I_{n}$	*p*+5
A×A	[ $1_{n}\;r\;c$ ]	[ $Z_{b}\;Z_{\mathrm{row}}\;Z_{\mathrm{col}}$ ]	$\sigma^{2}\;\Sigma_{c}(\rho_{c})⊗\Sigma_{r}(\rho_{r})$	*p*+7
A×Ae	[ $1_{n}\;r\;c$ ]	[ $Z_{b}\;Z_{\mathrm{row}}\;Z_{\mathrm{col}}$ ]	$\sigma^{2}\;\Sigma_{c}(\rho_{c})\;⊗\;\Sigma_{r}(\rho_{r})\;+\;\alpha I_{n}$	*p*+8
TPS	[ $X_{b}\;X_{s}$ ]	[ $Z_{\mathrm{row}}\;Z_{\mathrm{col}}\;Z_{s}$ ]	$\sigma^{2}\;I_{n}$	10

The total number of variance parameters includes the two variance parameters for the additive and non-additive genetic effects. *p* denotes the number of block terms, with value 1 or 2.

The baseline model is a simple independent error model with no modelling of spatial dependence. It contains a constant term ( $1_{n}$ ) as a fixed model term. In addition to the random genetic term, it has IID random terms to account for blocking effects (with design matrix $Z_{b}$ ) and row and column effects (with design matrices $Z_{\mathrm{row}}$ and $Z_{\mathrm{col}}$ ). We note that in the few cases where blocking is present in two directions, separate IID blocking terms are fitted for each direction.

The A×A model adds a number of terms to the baseline model. The fixed model includes linear trends in the row and column directions (denoted r and c in **Table 1**) to protect against this form of non-stationarity. The variance model for the plot errors takes the form $\sigma^{2}\;\Sigma_{c}(\rho_{c})⊗\Sigma_{r}(\rho_{r})$ which is a scaled direct product of separate autoregressive processes of order 1 (AR1) in the column and row directions. In each direction, this models a pattern of exponential decay as the lag between plots increases, where $\rho_{c}$ and $\rho_{r}$ are the lag 1 correlations in the column and row directions, respectively.

The A×Ae model adds an independent error term to the A×A variance model for the plot errors. The parameter α (**Table 1**) is the ratio of independent error variance to spatial process variance. We note that the independent error term has been widely referred to as the “nugget”, see for example, Gilmour et al. (1997); Piepho et al. (2015); Rodríguez-Álvarez et al. (2017); Velazco et al. (2017); Rodríguez-Álvarez et al. (2018). Use of the term nugget has it’s origins in geo-statistics so that our preference is to refer to the set of ‘independent error’ effects and their associated variance because this reflects their derivation from the errors-in-variables model of Besag (1977).

The TPS model takes a slightly different form, following that used by Velazco et al. (2017). The fixed part of the TPS consists of bilinear trend across the lattice surface, that is, $X_{s}=[1_{n}\left|r\right|c|r:c]$ here : indicates the interaction operator. Block terms are included in the fixed model, with design matrix $Z_{b}$ being relabeled as $X_{b}$ to indicate their status as a fixed model term(s). The random part of the model includes the genetic effects and IID terms for random row and column effects, plus the random part of the TPS. The latter is indicated by $Z_{s}$ and has 5 associated variance parameters. Details are given in the Appendix.

**Table 1** also counts the number of variance parameters associated with each model. The two genetic parameters have been added into this count in each case. The number of block terms, usually 1 but occasionally 2, is enumerated by *p*. We can see that a similar number of variance parameters is available across the AR and TPS approaches.

## 4 Empirical study

### 4.1 Fitted models

We analysed each trial using the A×A, A×Ae, TPS and baseline models. For the TPS model, we specified 6 and 19 (25) equally spaced knots for the row and column directions for lentils (field peas). This follows the approach of Velazco et al. (2017) and represents approximately 1 knot per two rows or columns. Example code to fit each model in ASReml-R is provided in the **Supplementary File** accompanying this paper.

### 4.2 Comparing the LMM

#### 4.2.1 Akaike information criteria

In the empirical study we have compared the four models using the AIC of Verbyla (2019) which can be used for models that have different sets of fixed terms and have been fitted using the REML method of variance parameter estimation. This gives an indication of the fit of the model to the data, with an adjustment for the number of variance parameters fitted. We have used the AIC to evaluate the difference in fit among the four models and to identify the best fitting model (lowest AIC) for each trial.

#### 4.2.2 Predicted genetic effects

A main objective in plant breeding field trials is to evaluate the best linear unbiased predictors (BLUPs) of the genetic effects for the set of test entries as these inform selection decisions. We note that in practice we use the empirical BLUPS (e-BLUPs) which are formed using the REML estimates of the variance parameters. A property of BLUP is that the correlation between the true underlying set of effects and the set of predicted effects is maximised. For the genetic effects, this is only true if the variance structure of the non-genetic effects is as close as possible to the true underlying structure. We therefore expect e-BLUPs from well-fitting models (judged by AIC) to be better for selection than those from poorer models (which will have different non-genetic variance structures). For models including genetic-relatedness, we need to consider both the additive and total genetic effects. Generally speaking, in inbred crops the e-BLUPs of the additive genetic effects are used for gene pool development while the e-BLUPs of the total genetic effects are used to select individuals to advance through the breeding program.

In Sections 5.3.1 and 5.3.2 we consider correlation and mis-classification measures based on the e-BLUPs of the additive and total genetic effects for each model and those of the best fitting model for each trial. In Section 5.3 we consider the correlation between the e-BLUPs of the additive and total genetic effects for each model and those of the best fitting model for each trial, mis-classification of entries for selection and distance between the e-BLUPs of the additive and total genetic effects for each model and those of the best fitting model for each trial. In Section 5.3.3 we use the distance measure of Martin (1990) to compare two models in terms of the *closeness* between their respective sets of estimates. For models 1 and 2 in a pair, the distance measure *d* is defined to be the sum of the squares of the differences between the individual estimates under the two models. For the predicted additive genetic effects say, *d* has the algebraic form


$$d=\sum_{i=1}^{m}{\left({\tilde{u}}_{a_{1_{i}}}-{\tilde{u}}_{a_{2_{i}}}\right)}^{2}$$

where ${\tilde{u}}_{ak_{i}}$ is the predicted additive effect for individual i under model *k, k* =1,2 and *m* is the number of individuals with data. Similarly for the total genetic effects. In Section 5.3.3 we also consider the distance measure for the subset of 74 trials where the A×Ae model (best performing of the three stochastic models) was the best fitting model. In this case, model 1 is the A×Ae model and model 2 is either the A×A, TPS or the baseline model. Likewise for the subset of 15 trials where the TPS model (only non-stochastic model) was the best fitting model. In this case, model 1 is the TPS model and model 2 is one of the three stochastic models.

## 5 Results

We have compared the A×A, A×Ae, TPS and baseline models based on iterations and convergence behaviour, goodness of fit using information criteria, and measures of discrepancy (mis-classification and distance) centred on the predicted genetic effects.

### 5.1 Iterations and convergence behaviour

For the A×A, TPS and baseline models there was no convergence failure for any trial. For A×Ae, one trial (P2HO19) failed to converge and was removed from any further calculation involving this model. The median number of iterations was 8, 9, 10 and 7 for the A×A, A×Ae, TPS and baseline models, respectively. The median time per iteration (in seconds) was 0.27, 0.45, 0.56 and 0.11 for this same order of models. The baseline model (no modelling of spatial dependence) converged in fewer iterations and in markedly less time than the other models. On the other hand, differences in run time between the AR and TPS models were negligible.

### 5.2 Information criteria

We have used the information criteria of Verbyla (2019) to compare the goodness of fit of the four models. **Table 2** gives the number and percentage of trials for which the A×A, A×Ae, TPS and baseline models achieved the lowest AIC and were consequently judged to be the *best* model for a trial. The total for each model is separated into the number of p-rep trials (S0, S1, P0) and the number of trials where the entries were mostly replicated (S2, P1, P2). Excluding the baseline model, a chi-squared goodness of fit test showed no difference in distribution across p-rep and replicated trials for the A×A, A×Ae and TPS models. The A×Ae model was judged to be the best model in 67.3% of trials (74) while the A×A and TPS models were each judged to be best in 13.6% (15) and 14.5% (16) trials, respectively. The baseline model was the best model in less than 5% of trials (5). Together the AR models were best in 80.9% of trials. The baseline model can be considered as part of both the ARIMA and TPS classes. The TPS approach would therefore lead to the best model in 19.1% of trials, and the AR approach to the best model in 85.5% of trials.

**Table 2:** Total number of trials where the A×A, A×Ae, TPS and baseline models were judged by AIC to be the best model.

Trial type	A×A	A×Ae	TPS	baseline	Total
p-rep	4	22	8	4	38
replicated	11	52	8	1	72
total	15	74	16	5	110
%	13.6	67.3	14.5	4.6	100

Percentages are also given. Totals are separated into the number of p-rep trials (S0, S1, P0) and trials in which the entries were mostly replicated (S2, P1, P2).

**Figure 1:**
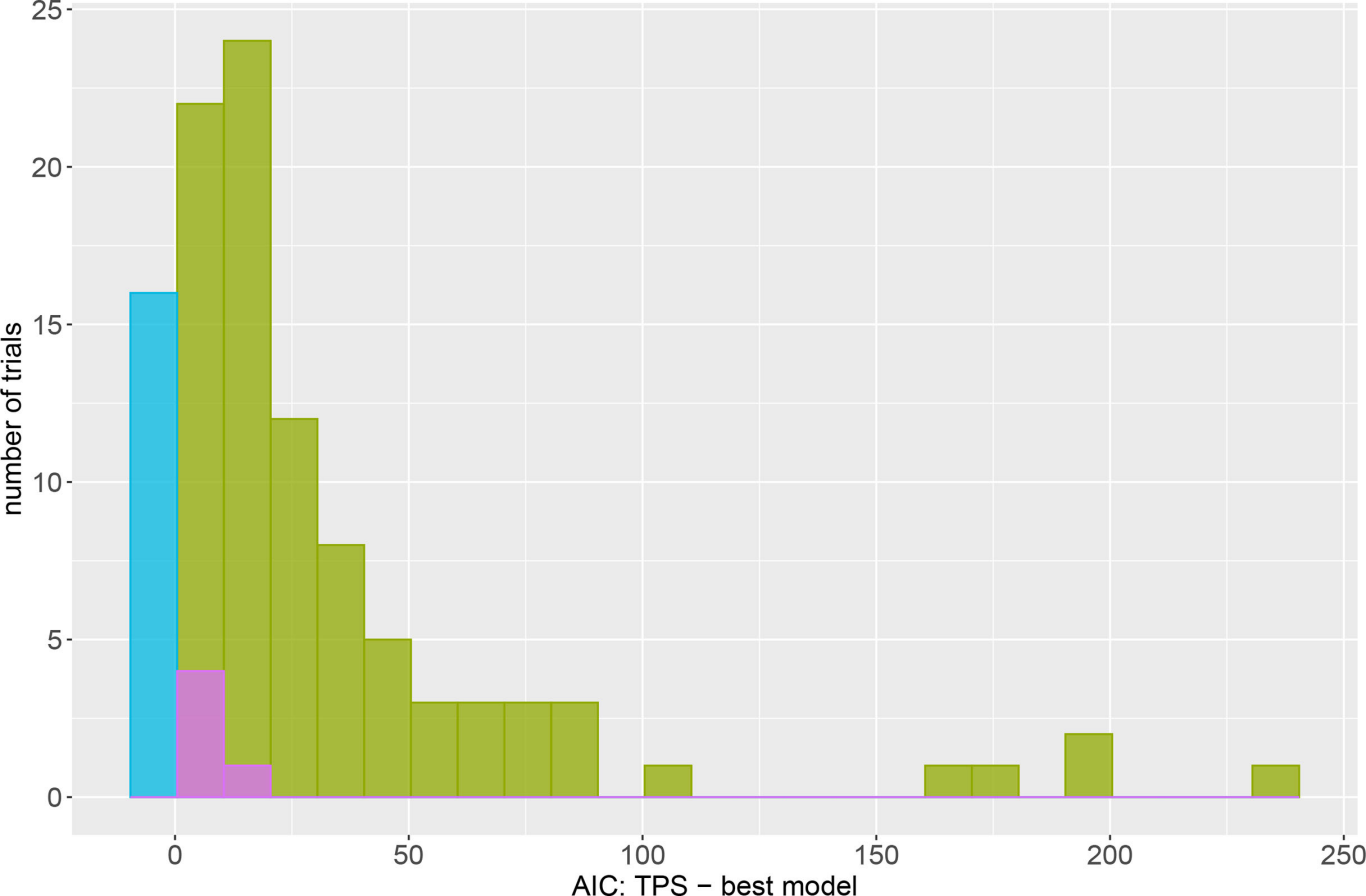
Histogram of the difference in AIC between the TPS model and the model with the lowest AIC within the set of four models. The blue bar at zero is for the trials where the TPS model was the best fitting model based on AIC. The green bars are for trials where the A×A or A×Ae model were the best model. Purple is for the five trials where the baseline model was the best model.

We are particularly interested in comparing the TPS model to the AR models. To gauge the amount by which the best model was superior to the TPS model, **Figure 1** is a histogram of the difference in AIC between the TPS and best model. The blue bar at zero is for the sixteen trials where the TPS model was itself judged best. The bars to the right of zero are for the 94 (85.5% of) trials where the A×A, A×Ae or baseline model was better than the TPS model (AR models indicated by green, baseline model by purple). On average, the best model was an improvement over TPS by 34.4 AIC units. In 40.4% of trials the TPS model was at least 25 AIC units worse than the best model, and in 19.1% of trials it was at least 50 AIC units worse than the best model. The baseline model (indicated by purple) was better than the TPS model by at most 15.5 AIC units. Conversely, the largest difference in AIC was for field pea trial P1GP20 for which the A×Ae model was better than the TPS model by 240.1 AIC units. In a final comparison we note that for the sixteen trials where the TPS model was the best fitting model, the most by which it was better than its closest competitor (the model with the next highest AIC) was by 9.6 AIC units. This demonstrates that in general, when the TPS model outperforms the AR models it does so by only a small amount compared to the AR models which can be the best fitting model by a substantial margin.

### 5.3 Predicted genetic effects

#### 5.3.1 Correlation with the best fitting model

The predicted genetic effects (additive and total) form the basis of selection decisions. For each trial we determined the e-BLUPs of the additive and total genetic effects for each of the four models A×A, A×Ae, TPS and baseline, together with their correlation with those of the best fitting model for that trial. **Table 3** gives the median and mean correlation for each model for both the additive and total effects. In each case the calculations have been undertaken excluding the set of trials for which the model under consideration was itself the best fitting model. For both types of effect (additive and total), the A×Ae model is most highly correlated with the best model across both summary statistics. The TPS model is less correlated with the best model than both AR models. For both sets of effects there is markedly lower correlation between the e-BLUPs of the best and baseline models when compared to the other three models.

**Table 3:** Median and mean correlation between the e-BLUPs of the additive and total genetic effects for the A×A, A×Ae, TPS and baseline models and those for the best model for each trial.

Effect type	Model	Number of trials	Median	Mean
additive	A × A	95	0.994	0.988
	A × Ae	35	0.998	0.996
	TPS	94	0.986	0.968
	baseline	105	0.969	0.936
total	A × A	95	0.991	0.982
	A × Ae	35	0.997	0.994
	TPS	94	0.983	0.965
	baseline	105	0.963	0.932

For a particular type of model, the summary statistics have been calculated excluding trials for which that model was the best fitting model. The number of trials in the subset of trials for each type of model is given in the third column.

#### 5.3.2 Mis-classification of entries

**Figure 2:**
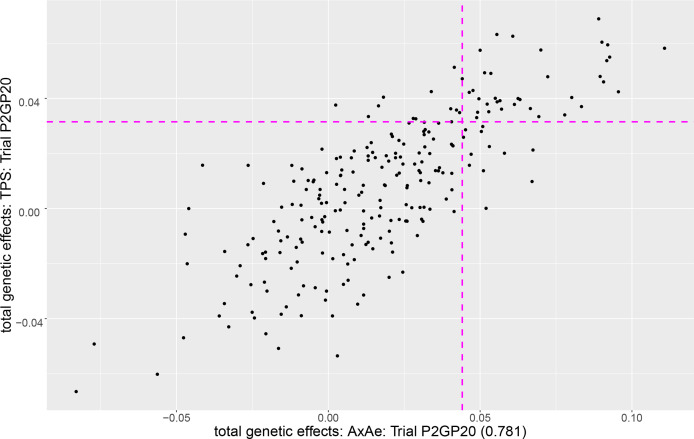
Plot of the total genetic effects for the TPS model against the A×Ae model for field pea trial P2GP20. The pink horizontal/vertical line separates out the top 20% of entries under the TPS/A×Ae model.

To demonstrate the discrepancy that can occur between the TPS and AR models, **Figure 2** is a plot of the predicted total effects for the TPS model against the A×Ae model (judged best) for field pea trial P2GP20 for which there were 241 entries. For this trial there was a drop of 200.02 in AIC units for A×Ae compared to TPS and a correlation of only 0.781 between the e-BLUPS (similar results for the additive genetic effects, not presented). The pink horizontal line separates out the top 20% of entries under the TPS model (49 entries above the line) while the pink vertical line separates out the top 20% of entries under the A×Ae model (49 entries to the right of the line). The top right quadrant contains entries that would be selected for progression under both models (37) while the entries in the bottom left quadrant would be discarded under both models (180). Conversely, the top left/bottom right quadrant is where the TPS model would select/reject while the A×Ae model would reject/select. In this case, 12 of the 49 entries selected under the A×Ae (best fitting) model, would not be selected under the TPS model, and vice versa. This represents a 24.5% discrepancy between the selection sets based on the A×Ae and TPS models (26.5% discrepancy for the set of additive genetic effects).

**Figure 3:**
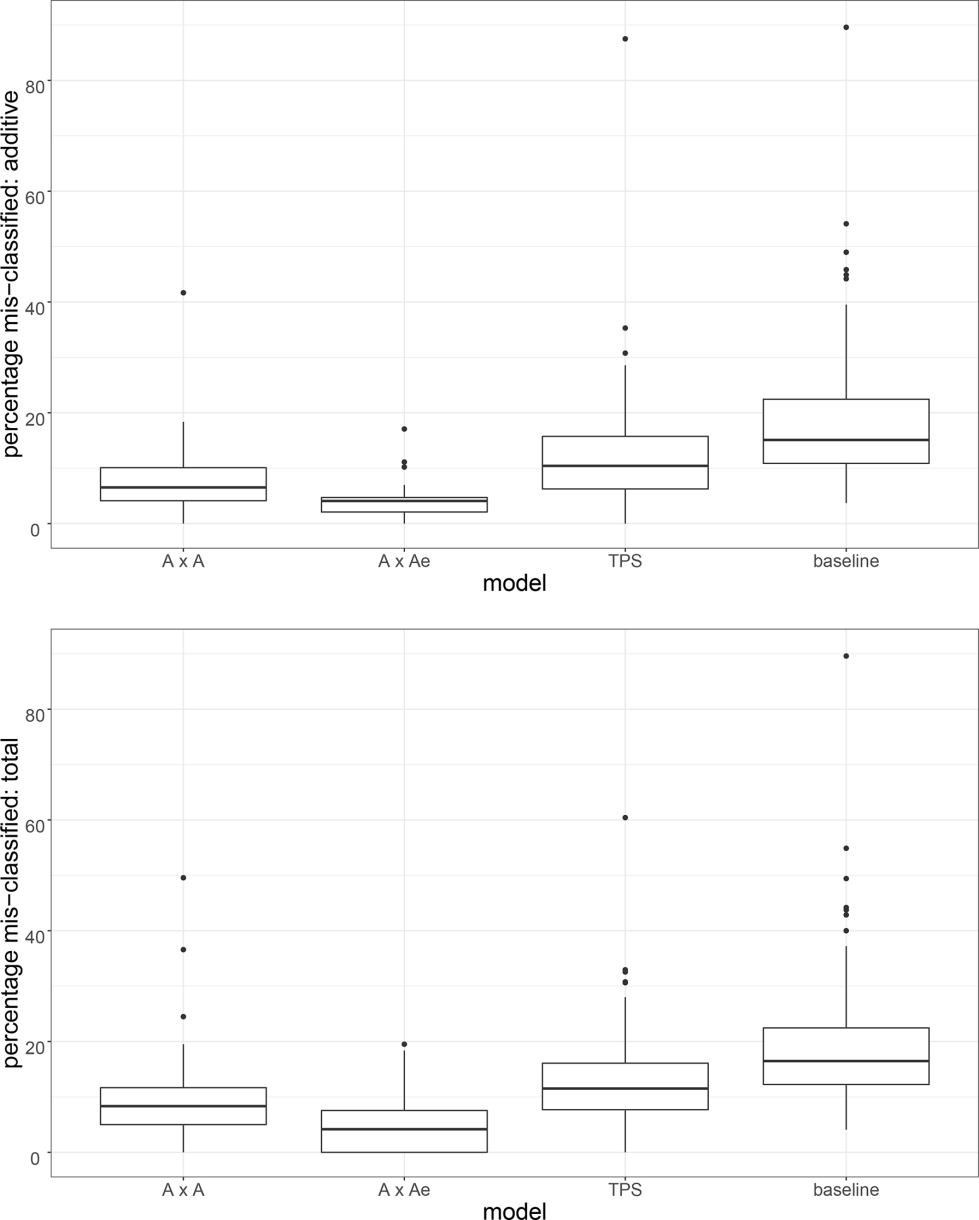
For the A×A, A×Ae, TPS and baseline models, a boxplot summary of the percentage of entries in the selection set (top ranking 20% of entries for each trial based on e-BLUP) that are mis-classified by that model when compared to the best model. Plots are for the additive effects (top plot) and total effects (bottom plot). For each model, trials for which that model was the best fitting model have been excluded from the summary.

This is just one example from the full set of 94 trials for which the TPS model was not the best model. For each of A×A, A×Ae, TPS and baseline, **Figure 3** presents a boxplot summary of the percentage of entries in the selection set (top ranking 20% of entries based on e-BLUP) that do not match the top 20% of entries for the best model and would therefore be mis-classified by that model. The plots are for the additive effects (top plot) and total effects (bottom plot). For each model, the distribution excludes the set of trials for which that model was the best fitting model. **Supplementary Table 2** contains the matching six-point summary for each model and type of effect.

We have chosen to consider median percentage mis-classification. This is to avoid the influence of a small number of trials for which there are large differences in the additive and/or non-additive variance parameter estimates, and consequently inflated values of mean percentage mis-classification between the best and other model. The results for median percentage mis-classification are consistent with those of **Table 3** for correlation between the predicted genetic effects. The A×Ae model has the lowest rate of mis-classification (4.08% for additive, 4.17% for total). The TPS model has a higher rate of mis-classification than both AR models (10.42% and 11.52% for TPS compared to 6.52% and 8.33% for A×A, and 4.08% and 4.17% for A×Ae). The rate of mis-classification is higher for the baseline model when compared to the other three models (15.09% for additive, 16.47% for total). There was no evidence of any difference in the rate of mis-classification for p-rep trials and rcb trials.

#### 5.3.3 Distance measure

The first six rows of **Table 4** give the median and mean distance measure of Martin (1990) (*d* of Section 4.2.2) for pairwise comparisons between the A×A, A×Ae, TPS and baseline models, for the sets of additive and total genetic effects and for the 109 trials in the data set (excluding trial P2HO19).

**Table 4:** The median and mean distance measure of Martin (1990) for pairwise comparisons between A×A, A×Ae, TPS and baseline models, for the sets of additive and total genetic effects and for all individuals in the data, rows 1 to 6.

Row	Comparison			Number of trials	Additive effects		Total effects	
					Median	Mean	Median	Mean
1	A×A	vs	A×Ae	109	0.03	0.09	0.06	0.32
2	A×A	vs	TPS	109	0.14	0.37	0.26	0.76
3	A×A	vs	baseline	109	0.17	0.49	0.33	0.99
4	A×Ae	vs	TPS	109	0.08	0.23	0.12	0.32
5	A×Ae	vs	baseline	109	0.18	0.42	0.28	0.65
6	TPS	vs	baseline	109	0.12	0.29	0.21	0.45
7	A×A	vs	A×Ae	74	0.03	0.10	0.07	0.43
8	TPS	vs	A×Ae	74	0.09	0.23	0.12	0.28
9	baseline	vs	A×Ae	74	0.23	0.46	0.30	0.62
10	A×A	vs	TPS	15	0.09	0.28	0.21	0.55
11	A×Ae	vs	TPS	15	0.05	0.15	0.07	0.35
12	baseline	vs	TPS	15	0.13	0.45	0.29	0.77

For each comparison, the statistics have been constructed using the full set of 109 trials in the data set. Also presented are the median and mean distance between the A×Ae model and the A×A, TPS and baseline models for the set of 74 where the A×Ae model was judged by AIC to be the best model, rows 7 to 9. Rows 10 to 12 present the median and mean distance between the TPS model and the A×A, A×Ae and baseline models for the set of 15 trials where the TPS model was judged to be the best model.

The A×A and A×Ae models are closer to each other than they are to the TPS model in terms of both median and mean distance for the additive effects and median distance for the total effects. They are as close or closer to each other in terms of mean distance for the total effects. We note that for the comparison between A×A and A×Ae for the set of total effects, there was an outlying distance of 16.73 for field pea trial P0CY17. For this trial, the non-additive genetic effects of the A×A model correspond closely to the sum of the non-additive genetic effects and the non-genetic independent errors (averaged for individuals) of the A×Ae model, the better model for this trial. This has resulted in a very large distance between the total effects for the two models and a shift in the mean distance for this comparison. There is a matching rate of misclassification of 49.6% for the A×A model for this trial, see Section 5.3.2. This is the maximum percentage mis-classification for this model and set of effects (see **Figure 3** and **Supplementary Table 2**). The apparent confounding of non-additive genetic effects and independent residual error for P0CY17 does not appear to have occurred to the same extent for other trials.

Rows 7 to 9 of **Table 4** allow us to directly compare the closeness of the A×A, TPS and baseline models to the A×Ae model for the 74 trials where the A×Ae model was judged to be the best model. Likewise, rows 10 to 12 allow us to compare the closeness of the A×A, A×Ae and baseline models to the TPS model (the non-stochastic model) for the set of 15 trials where the TPS model was best. Considering median figures (for reasons highlighted above concerning field pea trial P0CY17), when the A×Ae model is the best model, the A×A model is closer than the TPS model for both sets of effects. When the TPS model is best, the A×Ae model is closer for both sets of effects, with greater discrepancy between the A×A and TPS models.

It is not possible to make direct comparisons between the figures in rows 7 to 9 and rows 10 to 12 in **Table 4** due to their dependence on the genetic variances which vary between trials (75 trials for A×Ae and 15 for TPS). On the other hand, it is possible to compare the rate of mis-classification of entries for selection (range = 0 - 100%). For the 74 trials where the A×Ae model was the best model, the median percentage mis-classification for the A×A model was 6.5% for the additive effects and 8.3% for the total effects, compared to higher rates of mis-classification for the TPS model, that is, 10.3% for the additive effects and 11.3% for the total effects. For the 15 trials where the TPS model was the best model, there were relatively low rates of mis-classification for the A×Ae model, that is, 4.5% for the additive effects and 6.7% for the total effects, with a higher rate of mis-classification for the A×A model, that is, 8.2% for the additive effects and 10.9% for the total effects. For both subsets of trials and both sets of effects, the median percentage mis-classification was highest for the baseline model.

## 6 Discussion

The AR and TPS methods represent alternative approaches for the analysis of plant breeding field trial data for trials in which the plots have been arranged as a rectangular lattice. The AR approach makes use of low order time series models and the class of separable lattice processes to model two-dimensional spatial covariance among the plot errors. Alternatively, the TPS approach models two-dimensional smooth trend across the trial area.

We have analysed 110 pulse breeding trials using the A×A, A×Ae and TPS models, as well as a baseline model representing no modelling of spatial covariance or smooth trend effects. The A×Ae was judged by AIC to be the best fitting model for almost 70% of trials, and together the AR models were the best fitting models in more than 80% of trials. Conversely, the TPS model was the best fitting model in less than 15% of trials. We judged the goodness of fit of the four models using the AIC of Verbyla (2019). An alternative to the AIC is the Bayesian Information Criteria (BIC), see also Verbyla (2019). However, the BIC is known to favour simpler models (models with less variance parameters) and in our study the TPS model was best in just 2/110 = 1.8% of trials based on BIC. We therefore chose to proceed with AIC as the measure of goodness of fit in our study. For measures associated with the predicted genetic effects, the AR models, and in particular the A×Ae model, generally outperformed the TPS model in terms of being most highly correlated and closely aligned with the best fitting model. Perhaps the most relevant measure for plant breeding programs is the percentage mis-classification of entries for selection for comparisons with the best fitting model. The TPS model had consistently higher rates of mis-classification than both the A×A and A×Ae models.

An important outcome of this study is the degree to which the AR models are superior to the TPS model, particularly the A×Ae model which was generally also an improvement over the (nested) A×A model. Critics of the AR models (Piepho et al., 2015; Rodríguez-Álvarez et al., 2017; Velazco et al., 2017; Rodríguez-Álvarez et al., 2018) describe the A×Ae model as being slow to fit and prone to problems with convergence. In our study, the A×Ae model failed to converge for just one in the full set of 110 trials, with negligible difference in run-time between the AR and TPS models. For plant breeding programs currently implementing the A×A model, this may motivate a greater use of the A×Ae model. On the other hand, the A×A model has been shown to be generally closely aligned with the A×Ae model and is therefore expected to perform well even in cases where the A×Ae model is a better fit for the data.

One major difference between the TPS and AR models is the presence of non-stationarity in the TPS model. This means that the variance and correlation patterns for the TPS model can change across the field trial area, and might be thought to give additional flexibility. However, the non-stationarity takes a pre-determined form, dependent on the spline design matrices, that seems incompatible with any likely underlying variance or correlation pattern. This may be one reason why the AR models generally performed better in our study.

The A×A and A×Ae models are just two from the wider class of ARIMA models. Consequently, in cases where the A×A or A×Ae models do not provide an adequate representation of the spatial dependence in the data, there is a wide range of alternative time series and other related models to choose from. For example, the equal-roots autoregressive model of Stringer et al. (2011) is likely to provide a better fit in the presence of competition effects. This highlights the flexibility and utility of autoregressive processes, and particularly the ARIMA class, in modelling spatial dependence.

Piepho et al. (2022) have recently carried out a detailed study of TPS models in the context of plant breeding trials. They note that the TPS model with second-order differencing implemented in SpATS and used here requires the introduction of correlation between random terms to ensure invariance to choice of the underlying eigenvector basis. The difficulty in fitting these correlation parameters may prompt practitioners to omit them, so our results are relevant in showing the performance of these models ignoring the extra parameters. Piepho et al. (2022) used their results as the motivation to consider TPS models with first-order differencing. Choice of an appropriate TPS model reintroduces a model selection step that the SpATS approach had been intended to avoid (Velazco et al., 2017). Given that the TPS approach does not avoid problems associated with model selection, and that in its simple use here it does not perform as well as the AR models in terms of selection of entries, we strongly recommend the continued use of time series models in field trial analysis.

The results of this study are significant, particularly for plant breeding programs seeking to implement the most efficient processes within their analysis and evaluation systems.

## Data availability statement

The data analyzed in this study is subject to the following licenses/restrictions: The datasets presented in this article are not readily available because they are owned by Agriculture Victoria and the Grains Research and Development Corporation. Requests to access these datasets should be directed to Garry Rosewarne, garry.rosewarne@agriculture.vic.gov.au.

## Author contributions

BC conceived the ideas and BG, SW and BC partnered in developing them. BG prepared the data, undertook the empirical study and prepared the first draft of the manuscript and SW provided critical review comments throughout the manuscript preparation. All authors contributed to general discussions and approved the final submitted version.

## Appendix A

The TPS model is the special case of model (1) in which spatial trend is modelled as a smooth two-dimensional function of the plot positions in both the row and column dimensions, represented as a combination of fixed and random spatial terms. The smooth trend component of the TPS model can be written algebraically as

$$g(r,c)=X_{s}\tau_{s}+Z_{s}u_{s}$$

where g(r,c) represents the smooth two-dimensional function. This function is an interaction between two one-dimensional cubic P-splines with a modified second-order differencing penalty. Let $r_{a}=r-\bar{r}1_{n}$ and $c_{a}=c-\bar{c}1_{n}$ be the *n*×1 vectors of centred row and column positions. In addition, let *k_r_* and *k_c_* be the number of knots in the row and column directions, respectively. In the row direction, the design matrix for a cubic P-spline with second-order differencing penalty can be written as [ $1_{n}r_{a}Z_{r}$ ] (Velazco et al., 2017). In this matrix, the first two terms represent linear trend in the row direction while $Z_{r}$ has *k_r_* columns and represents a basis for smooth non-linear trend in the same direction. Likewise for columns, with matrix [ $1_{n}c_{a}Z_{c}$ ]. Interacting these matrices results in composite matrices $X_{s}$ and $Z_{s}$ , where $X_{s}=[1_{n}\;|\;r_{a}\;|\;c_{a}\;|\;r_{a}:c_{a}]$ . The matrix $Z_{s}=[Z_{r}\;|\;c_{a}:Z_{r}\;|\;Z_{c}\;|\;r_{a}:Z_{c}\;|\;Z_{r}:Z_{c}]$ where $Z_{r}$ and $Z_{c}$ are as defined above and represent smooth trend over rows and columns, respectively, $c_{a}:Z_{r}$ represents linear trend in columns varying smoothly over rows, $r_{a}:Z_{c}$ represents linear trend in rows varying smoothly over columns and $Z_{r}:Z_{c}$ represents a smooth two-dimensional surface. In g(r,c) , $X_{s}\tau_{s}$ represents the unpenalised partition and the terms in $\tau_{s}$ are fitted as fixed model terms. Conversely, $Z_{s}u_{s}$ is the penalised partition, and the terms in $u_{s}$ that correspond to the five components in $Z_{s}$ are fitted with known diagonal variance matrices, each with its own variance component to be estimated (Rodríguez-Álvarez et al., 2018 for details). Finally, the sum of the fitted values from the full set of fixed and random terms gives the smooth spatial surface.

In theory, there is a choice of polynomial degree for B-spline basis underpinning the P-spline in each dimension, the order of differencing and the number of equally-spaced knots. In practice, a cubic B-spline basis with a modified second-order differencing penalty in each direction is the usual choice. Velazco et al. (2017) suggested 1 knot per 2 units in each direction for modelling spatial trend in field trials and give full details of the models.

## References

[B1] Bartlett M. S. (1978). Nearest neighbour models in the analysis of field experiments. J. R. Stat. Soc. Ser. B 40, 147–158. doi: 10.1111/j.2517-6161.1978.tb01657.x

[B2] Beeck C. P. Cowling W. A. Smith A. B. Cullis B. R. (2010). Analysis of yield and oil from a series of canola breeding trials. part 1. fitting factor analytic mixed models with pedigree information. Genome 53, 992–1001. doi: 10.1139/G10-051 21076515

[B3] Besag J. (1977). Errors-in-variables estimation for gaussian lattice schemes. J. R. Stat. Soc. Ser. B Methodol. 39, 73–78. doi: 10.1111/j.2517-6161.1977.tb01607.x

[B4] Besag J. Kempton R. A. (1986). Statistical analysis of field experiments using neighbouring plots. Biometrics 42, 231–251. doi: 10.2307/2531047

[B5] Box J. F. (1978). R. a. Fisher, the life of a scientist (New York: Wiley).

[B6] Butler D. G. (2013). On the optimal design of experiments under the linear mixed model (Queensland, Australia:: School of Mathematics and Physics, The University of Queensland).

[B7] Butler D. G. Cullis B. R. Gilmour A. R. Gogel B. J. Thompson R. (2018). ASReml-r reference manual version 4. VSN international.

[B8] Cullis B. R. Gleeson A. C. (1991). Spatial analysis of field experiments - an extension to two dimensions. Biometrics 47, 1449–1460. doi: 10.2307/2532398

[B9] Cullis B. R. Smith A. B. Cocks N. A. Butler D. G. (2020). The design of early-stage plant breeding trials using genetic relatedness. journal of agricultural. Biol. Environ. Stat 25, 553–578. doi: 10.1007/s13253-020-00403-5

[B10] Cullis B. R. Smith A. B. Coombes N. E. (2006). On the design of early generation variety trials with correlated data. journal of agricultural. Biol. Environ. Stat 11 (4), 381–393. doi: 10.1198/108571106X154443

[B11] De Faveri J. Verbyla A. Cullis B. Pitchford W. Thompson R. (2017). Residual variance–covariance modelling in analysis of multivariate data from variety selection trials. J. Agricult. Biol. Environ. Stat 22, 1–22. doi: 10.1007/s13253-016-0267-0

[B12] Ganesalingam A. Smith A. B. Beeck C. Cowling W. Thompson R. Cullis B. (2012). A bivariate mixed model approach for the analysis of plant survival data. Euphytica 190, 371–383. doi: 10.1007/s10681-012-0791-0

[B13] Gilmour A. R. Cullis B. R. Verbyla A. P. (1997). Accounting for natural and extraneous variation in the analysis of field experiments. journal of agricultural. Biol. Environ. Stat 2, 269–273. doi: 10.2307/1400446

[B14] Gleeson A. C. Cullis B. R. (1987). Residual maximum likelihood (REML) estimation of a neighbour model for field experiments. Biometrics 43, 277–288. doi: 10.2307/2531812

[B15] Green P. J. (1985). Linear models for field trials, smoothing and cross-validation. Biometrika 72, 527–537. doi: 10.1093/biomet/72.3.527

[B16] Green P. J. Jennison C. Seheult A. H. (1985). Analysis of field experiments by least squares smoothing. J. R. Stat. Soc. Ser. B 47, 299–315. doi: 10.1111/j.2517-6161.1985.tb01358.x

[B17] Hunt C. Hayes B. Eeuwijk F. Mace E. Jordan D. (2020). Multi-environment analysis of sorghum breeding trials using additive and dominance genomic relationships. Theor. Appl. Genet. 133 (3), 1009–1018. doi: 10.1007/s00122-019-03526-7 31907563

[B18] Laslett G. M. (1994). Kriging and splines: An empirical comparison of their predictive performance in some applications. J. Am. Stat. Assoc. 89, 391–400. doi: 10.1080/01621459.1994.10476759

[B19] Martin R. J. (1990). The use of time-series models and methods in the analysis of agricultural field trials. Commun. Stat - Theory Methods 19, 55–81. doi: 10.1080/03610929008830187

[B20] Norman A. Taylor J. Edwards J. Kuchel H. (2018). Optimising genomic selection in wheat: Effect of marker density, population size and population structure on prediction accuracy. G3-Genes Genomes Genet. 8, g3.200311. doi: 10.1534/g3.118.200311 PMC6118301 29970398

[B21] Oakey H. Cullis B. Thompson R. Comadran J. Halpin C. Waugh R. (2016). Genomic selection in multi-environment crop trials. G3: Genes. Genomes Genet. 6, 1313–1326. doi: 10.1534/g3.116.027524 PMC4856083 26976443

[B22] Oakey H. Verbyla A. Pitchford W. Cullis B. Kuchel H. (2006). Joint modeling of additive and non-additive genetic line effects in single field trials. Theor. Appl. Genet. 113, 809–819. doi: 10.1007/s00122-006-0333-z 16896718

[B23] Papadakis J. S. (1937). Methode statistique pour des experiences sur champ. bulletin scientifique (Grece: Institut d’Amelioration des Plantes a Thessaloniki).

[B24] Patterson H. D. Thompson R. (1971). Recovery of interblock information when block sizes are unequal. Biometrika 31, 100–109. doi: 10.2307/2334389

[B25] Piepho H.-P. Boer M. Williams E. (2022). Two-dimensional p-spline smoothing for spatial analysis of plant breeding trials. Biom. J. 64 (5), 1–23. doi: 10.1002/bimj.202100212 35692062

[B26] Piepho H.-P. Möhring J. Pflugfelder M. Hermann W. Williams E. (2015). Problems in parameter estimation for power and ar(1) models of spatial correlation in designed field experiments. Commun. Biom. Crop Sci. 10, 3–16. doi: 10.1017/S0021859614000823

[B27] R Development Core Team (2020). R: A language and environment for statistical computing (Vienna, Austria: R Foundation for Statistical Computing), ISBN: ISBN 3-900051-07-0.

[B28] Rodríguez-Álvarez M. X. Boer M. P. van Eeuwijk F. A. Eilers P. H. (2017). Correcting for spatial heterogeneity in plant breeding experiments with p-splines. Spatial Stat 23, 52–71. doi: 10.1016/j.spasta.2017.10.003

[B29] Rodríguez-Álvarez M. X. Boer M. P. van Eeuwijk F. A. Eilers P. H. C. (2018). Modelling spatial trends in sorghum breeding field trials using a two-dimensional p-spline mixed model. Spatial Stat 23, 52–71. doi: 10.1016/j.spasta.2017.10.003 PMC5487705 28374049

[B30] Smith A. Cullis B. Thompson R. (2001). Analyzing variety by environment data using multiplicative mixed models and adjustments for spatial field trend. Biometrics 57, 1138–1147. doi: 10.1111/j.0006-341X.2001.01138.x 11764254

[B31] Stringer J. Cullis B. Thompson R. (2011). Joint modeling of spatial variability and within-row interplot competition to increase the efficiency of plant improvement. J. Agric. Biol. Environ. Stat 16, 269–281. doi: 10.1007/s13253-010-0051-5

[B32] Tolhurst D. J. Mathews K. L. Smith A. B. Cullis B. R. (2019). Genomic selection in multi-environment plant breeding trials using a factor analytic linear mixed model. J. Anim. Breed. Genet. 136, 279–300. doi: 10.1111/jbg.12404 31247682

[B33] Velazco J. G. Rodríguez-Álvarez M. X. Boer M. P. Jordan D. R. Eilers P. H. Malosetti M.. (2017). Modelling spatial trends in sorghum breeding field trials using a two-dimensional p-spline mixed model. Theor. Appl. Genet. 130, 1375–1392. doi: 10.1007/s00122-017-2894-4 28374049 PMC5487705

[B34] Verbyla A. P. (2019). A note on model selection using information criteria for general linear models estimated using REML. Aust. New Z. J. Stat 61, 39–50. doi: 10.1111/anzs.12254

[B501] Welham S. J. (2019). TPSbits package Available at: https://mmade.org/tpsbits. (Last accessed on 2022-04-04).

[B35] Wilkinson G. N. Eckert S. R. Hancock T. W. Mayo O. (1983). Nearest neighbour (NN) analysis of field experiments (with discussion). J. R. Stat. Soc. Ser. B 45, 151–211. doi: 10.1111/j.2517-6161.1983.tb01240.x

