# Supplementary material for: Empirical comparison of time series models and tensor product penalised splines for modelling spatial dependence in plant breeding field trials
Source: Front Plant Sci. 2023 Jan 18;13:1021143. doi: 10.3389/fpls.2022.1021143 (PMC9987337; doi:10.3389/fpls.2022.1021143)
Supplement: Supplementary file 1 [file DataSheet_1.pdf]

Supplementary Material

Supplementary Table 1

Summary of the full set of 110 trials in the 2016 - 2020 combined lentil and field pea data set, ordered as trial in year in stage in crop. Information includes the trial name and stage, the number of rows in the trial layout (nrow), the total number of genotypes (ngeno) and the number of individuals with 1 (1r) and 2 or more (≥2r) replicates, the trial mean yield (mean) and the number of missing data observations (miss).

Trial	Stage	nrow	ngeno	1r	≥2r	mean	miss	Trial	Stage	nrow	ngeno	1r	≥2r	mean	miss
L1AHO16	S0	14	157	153	4	3.11	1	P0HO19	P0	76	762	612	150	1.82	5
LM1AHO16	S0	18	202	199	3	3.82	5	P0BE20	P0	70	636	435	201	2.05	4
L1AHO17	S0	55	527	416	111	2.58	1	P0HO20	P0	70	662	485	177	3.74	5
L1BHO17	S0	8	72	50	22	1.71	0	P1BA17	P1	50	299	0	299	1.35	1
LM1AHO17	S0	61	590	467	123	2.24	4	P1GP17	P1	50	299	0	299	2.42	1
LM1BHO17	S0	6	45	23	22	1.50	0	P1HO17	P1	50	299	0	299	3.33	1
L1AHO18	S0	80	788	616	172	1.48	0	P1WW17	P1	50	299	0	299	1.57	4
L1BHO18	S0	14	128	93	35	0.87	2	P1BA18	P1	70	409	1	408	0.82	1
LM1AHO18	S0	53	521	406	115	2.29	5	P1HO18	P1	70	410	4	406	2.50	3
LM1BHO18	S0	10	88	58	30	1.66	3	P1SC18	P1	70	410	2	408	1.85	0
LM0HO19	S0	34	332	262	70	1.37	0	P1BA19	P1	70	421	6	415	0.70	4
L0HO19	S0	57	567	461	106	1.47	2	P1GP19	P1	70	421	10	411	0.33	6
LGS0HO19	S0	48	202	7	195	1.42	0	P1HO19	P1	70	421	11	410	1.74	5
L0HO20	S0	54	528	415	113	2.46	2	P1WW19	P1	70	420	4	416	0.34	0
LM0HO20	S0	36	346	260	86	2.09	4	P1BA20	P1	70	421	2	419	2.17	2
L1RHO17	S1	24	224	169	55	3.08	0	P1GP20	P1	70	422	4	418	0.90	13
L1RMH17	S1	24	224	169	55	1.65	0	P1HO20	P1	70	421	4	417	3.65	3
L1RML17	S1	24	224	169	55	1.85	0	P1WW20	P1	70	422	4	418	2.51	1
L1RCY18	S1	50	498	398	100	0.22	1	P2AR18	P2	40	240	0	240	0.76	0
L1RHO18	S1	50	497	396	101	1.68	1	P2BA18	P2	40	240	0	240	0.83	0
L1RMH18	S1	50	499	398	101	0.98	1	P2CY18	P2	40	240	0	240	0.33	0
L1RML18	S1	50	498	397	101	1.14	2	P2GP18	P2	40	240	0	240	1.70	0
L1RHO19	S1	50	488	386	102	1.59	1	P2HO18	P2	40	238	19	219	2.02	19
L1RMH19	S1	50	489	386	103	1.09	0	P2KI18	P2	40	238	0	238	1.68	0
L1RML19	S1	50	489	386	103	0.99	2	P2M118	P2	40	239	2	237	0.50	9
L1RHO20	S1	42	404	305	99	2.47	0	P2SC18	P2	40	237	0	237	1.92	0
L1RMH20	S1	42	401	303	98	2.66	1	P2SN18	P2	40	237	0	237	0.74	2
L1RML20	S1	42	404	305	99	0.80	1	P2WW18	P2	40	240	0	240	1.14	0
L2RCY18	S2	32	192	0	192	0.39	0	P2AR19	P2	40	245	11	234	0.42	0
L2RHO18	S2	32	192	0	192	1.87	2	P2BA19	P2	40	245	12	233	0.88	2
L2RKD18	S2	32	192	0	192	0.61	0	P2BE19	P2	40	245	13	232	1.33	0
L2RMH18	S2	32	192	0	192	1.09	0	P2CY19	P2	40	245	13	232	2.97	1
L2RML18	S2	32	192	12	180	1.30	15	P2GP19	P2	40	245	11	234	0.24	1
L2RMY18	S2	32	192	0	192	0.46	0	P2HO19	P2	40	241	7	234	1.87	7
L2RBE19	S2	38	228	0	228	0.82	2	P2KA19	P2	40	245	11	234	1.44	0
L2RCY19	S2	38	230	5	225	1.53	0	P2KI19	P2	40	243	7	236	1.63	0
L2RHO19	S2	38	228	1	227	1.37	62	P2MI19	P2	40	244	9	235	1.00	6
L2RKD19	S2	38	230	4	226	0.98	1	P2SC19	P2	40	245	11	234	1.39	5
L2RMH19	S2	38	230	5	225	1.25	0	P2SL19	P2	40	246	13	233	1.67	0
L2RML19	S2	38	230	5	225	0.95	1	P2WW19	P2	40	244	10	234	0.38	0
L2RMY19	S2	38	228	1	227	1.41	0	P2YE19	P2	40	244	10	234	1.26	0

Summary of the full set of 110 trials in the 2016 - 2020 combined lentil and field pea data set, ordered as trial in year in stage in crop. Information includes the trial name and stage, the number of rows in the trial layout (nrow), the total number of genotypes (ngeno) and the number of individuals with 1 (1r) and 2 or more (≥2r) replicates, the trial mean yield (mean) and the number of missing data observations (miss).

Trial	Stage	nrow	ngeno	1r	≥2r	mean	nmiss	Trial	Stage	nrow	ngeno	1r	≥2r	mean	miss
L2BE20	S2	36	212	0	212	1.85	0	P2AR20	P2	40	240	3	237	2.69	0
L2CY20	S2	36	212	0	212	2.17	1	P2BA20	P2	40	242	5	237	2.33	1
L2HO20	S2	36	212	0	212	2.64	0	P2BE20	P2	40	242	5	237	2.02	1
L2KD20	S2	36	212	0	212	1.16	0	P2CY20	P2	40	241	7	234	2.62	2
L2MH20	S2	36	212	0	212	0.90	1	P2GP20	P2	40	241	7	234	0.83	1
L2ML20	S2	36	212	0	212	0.90	0	P2HO20	P2	40	243	8	235	3.79	0
L2MY20	S2	36	212	0	212	2.63	1	P2KA20	P2	40	241	7	234	1.54	0
P0HO16	P0	42	404	311	93	3.19	5	P2KI20	P2	40	238	4	234	2.56	3
P0BE17	P0	50	568	536	32	0.89	0	P2MI20	P2	40	238	4	234	0.96	0
P0CY17	P0	50	562	524	38	1.92	12	P2SC20	P2	40	239	4	235	1.98	2
P0HO17	P0	88	877	701	176	3.03	4	P2SL20	P2	40	242	8	234	1.80	2
P0SL17	P0	50	554	508	46	1.44	1	P2SN20	P2	40	240	8	232	0.81	0
P0HO18	P0	80	797	637	160	2.34	2	P2WW20	P2	40	243	11	232	2.97	2
P0BE19	P0	76	758	609	149	1.43	7	P2YE20	P2	40	239	6	233	2.79	0

Supplementary Table 2

For the A×A, A×Ae, TPS and baseline models, a six-point summary of the percentage of entries in the selection set (top ranking 20% of entries based on e-BLUP) that do not match the top 20% of entries for the best model. For each model and set of effects (additive and total) the calculations have been undertaken excluding the set of trials for which that model was the best model.

	additive effects				total effects			
	A×A	A×Ae	TPS	baseline	A×A	A×Ae	TPS	baseline
Minimum	0.00	0.00	0.00	3.70	0.00	0.00	0.00	4.08
1st Quartile	4.12	2.08	6.25	10.87	5.00	0.00	7.69	12.24
Median	6.52	4.08	10.42	15.09	8.33	4.17	11.52	16.47
Mean	7.40	4.01	12.47	17.67	9.26	5.28	13.18	19.26
3rd Quartile	10.08	4.71	15.75	22.45	11.67	7.55	16.09	22.45
Maximum	41.67	17.07	87.50	89.58	49.56	19.51	60.42	89.58

Supplementary code

```
######################################################################################## ---
#calls to asreml() for the baseline, AxA, AxAe and TPS models for a trial with
#blocking in two directions ----

#trial.df - data frame
#yield - response variable
#Gkeep - factor corresponding to those individuals with pedigree information
#Ainv - inverse numerator relationship matrix
#RowRep - factor defining row replicates
#ColRep - factor defining column replicates
#Row - factor for rows
#Column - factor for columns

#baseline model ----
base.asr <- asreml(yield ~ 1,
               random = ~ vm(GKeep, Ainv) + ide(GKeep) + RowRep + ColRep + Row + Column,
               data= trial.df, na.action = na.method(x='include'), maxiter = 30)

#AxA model ----
axa.asr <- asreml(yield ~ 1 + lin(Column) + lin(Row),
               random = ~ vm(GKeep, Ainv) + ide(GKeep) + RowRep + ColRep + Row + Column,
               residual = ~ ar1(Column):ar1(Row),
               data= trial.df, na.action = na.method(x='include'), maxiter = 30)

#AxAe model ----
axae.asr <- asreml(yield ~ 1 + lin(Column) + lin(Row),
               random = ~ vm(GKeep, Ainv) + ide(GKeep) + RowRep + ColRep + Row + Column +
               units,
               residual = ~ ar1(Column):ar1(Row),
               data= trial.df, na.action = na.method(x='include'), maxiter = 30)

#TPS model ----
#degree = 3 (cubic smoothing spline) with pord = 2 (second order differencing) ...
knots.col <-  6
knots.row <-  19
nsegcol <- knots.col - 1
nsegrow <- knots.row - 1

trial.df$row <- as.numeric(trial.df$Row)
trial.df$col <- as.numeric(trial.df$Column)

#get structures for TPS model fit ----
TPXZ <- tpsmmb("col", "row", trial.df, nsegments=c(nsegcol, nsegrow))
BcZ1.df <- TPXZ$BcZ.df
BrZ1.df <- TPXZ$BrZ.df
BcrZ1.df <- TPXZ$BcrZ.df

#run TPS model ----
TPS.asr <- asreml(yield ~ 1 + TP.CR.2 + TP.CR.3 + TP.CR.4 + ColRep + RowRep,
           random = ~ vm(GKeep, Ainv) + ide(GKeep) + Row + Column +
                 TP.C.1:mbf(TP.row) + TP.C.2:mbf(TP.row) +
                 TP.R.1:mbf(TP.col) + TP.R.2:mbf(TP.col) + mbf(TP.CxR),
                 mbf = list(TP.col=list(key=c("TP.col","TP.col"),cov="BcZ1.df"),
                 TP.row=list(key=c("TP.row","TP.row"),cov="BrZ1.df"),
                 TP.CxR=list(key=c("TP.CxR","TP.CxR"),cov="BcrZ1.df")),
                 data = TPXZ$data, na.action = na.method(x='include'), maxiter = 30)
```

```
####################################################################################### ---
#end of code ----
```
